# West Nile Virus Seroprevalence in the Greek Population in 2013: A Nationwide Cross-Sectional Survey

**DOI:** 10.1371/journal.pone.0143803

**Published:** 2015-11-25

**Authors:** Christos Hadjichristodoulou, Spyros Pournaras, Maria Mavrouli, Andriani Marka, Persefoni Tserkezou, Agoritsa Baka, Charalambos Billinis, Antonios Katsioulis, Anna Psaroulaki, Anna Papa, Nikos Papadopoulos, Zissis Mamuris, Athanasios Tsakris, Jenny Kremastinou, MALWEST Project

**Affiliations:** 1 Department of Hygiene and Epidemiology, Faculty of Medicine, University of Thessaly, Larissa, Greece; 2 Department of Microbiology, Faculty of Medicine, National and Kapodistrian University of Athens, Athens, Greece; 3 Hellenic Centre for Disease Control & Prevention (KEELPNO), Athens, Greece; 4 Laboratory of Microbiology and Parasitology, Faculty of Veterinary Medicine, University of Thessaly, Karditsa, Greece; 5 Laboratory of Bacteriology, Parasitology, Zoonoses and Geogrphical Medicine, School of Medicine, University of Crete, Heraklion, Greece; 6 National Reference Center for Arboviruses, Department of Microbiology, School of Medicine, Aristotle University of Thessaloniki, Thessaloniki, Greece; 7 Laboratory of Entomology and Agricultural Zoology, School of Agricultural Sciences, Department of Agriculture Crop Production and Rural Environment, University of Thessaly, Volos, Greece; 8 Department of Biochemistry and Biotechnology, University of Thessaly, Larissa, Greece; Centre d'Immunologie de Marseille-Luminy, CNRS-Inserm, FRANCE

## Abstract

Cases of West Nile Virus (WNV) disease were recorded for three consecutive years in Greece following the year 2010 outbreak. A cross-sectional serologic survey was conducted to estimate the WNV seroprevalence and assess the ratio of infection to neuroinvasive disease. A stratified left-over sampling methodology was used including age and residence strata. A total of 3,962 serum samples was collected and tested for WNV Immunoglobulin G (IgG) antibodies by Enzyme–Linked Immunosorbent Assay (ELISA). All positive samples were further tested by Plaque Reduction Neutralization Test (PRNT) and WNV Immunoglobulin M (IgM) antibodies. WNV IgG antibodies were detected in 82 samples and 61 were also positive in PRNT representing a weighted seroprevalence of 2.1% (95% C.I.: 1.7–2.6) and 1.5% (95% C.I.: 1.2–2.0), respectively. Multivariable analysis showed that seroprevalence was associated with age and residence. The overall ratio of neuroinvasive disease to infected persons was estimated at 1:376 (95% C.I.: 1:421–1:338), while the elderly people had the highest ratio. This nationwide study provided valuable data regarding the epidemiology of WNV in Greece based on the fact that elderly people have higher risk of being both infected and having severe disease.

## Introduction

WNV is considered to be one of the most important emerging arboviruses in recent years involving birds and by using *Culex* mosquitoes in its transmission cycle [1]. The recent spread of the disease in Europe and in the United States of America (USA) where many laboratory-confirmed human cases and deaths raise concerns about the future behavior of WNV. Outbreaks with severe symptoms have been reported in Europe (for example, Romania, Hungary, Italy, and Greece) in the last two decades [2–5].

Until 2004, the lineage 2 of WNV was never detected outside the African continent. However, in subsequent years, when the first detection of a strain of this lineage was reported from southeastern Hungary in 2004 [2], many reports of sporadic cases and outbreaks have occurred in various countries in Eastern Europe (Hungary, Russia, Romania), Southern Europe (Greece, Italy, Albania, Serbia) and the Middle East (Israel). One of the largest WNV disease outbreaks in Europe, also took place in Greece in the year 2010; thereafter, similar WNV cases occurred every year during the transmission period (June to September). The first cases were reported in northern Greece (central Macedonia) near the city of Thessaloniki; the area is characterized by many wetlands, rivers and lakes that serve as stopping areas for migratory birds during their migration from overwintering areas in Africa to breeding sites in northern Europe and vice versa.

Evidence from mosquitoes, birds and blood donors demonstrated that the epidemic was caused by WNV lineage 2 [6–8]. In subsequent years, the disease further spread both southwards and eastwards, and cases of occurrence were reported even in the highly-populated Greek capital city of Athens. A total of 609 laboratory confirmed cases and 73 deaths were reported between the year 2010 and 2013, making this outbreak the largest of WNV disease in Europe [9–11]. Many equine cases of WNV disease were reported as well; a total of 83 cases were confirmed within year 2010 and 2013.

WNV is considered to be maintained in an enzootic cycle with wild and domestic birds being the main amplifying hosts as well as ornithophilic mosquitoes, especially of the *Culex* species, being the main vectors. Moreover, both local movements of resident birds and long-range travel of migratory birds may contribute to the spread of WNV [12]. Most human infections remain asymptomatic, as West Nile fever develops in ≈20% and West Nile Neuroinvasive Disease (WNND) in <1% of infected persons [13].

Exposure of humans to the virus may be considerably different when evaluating laboratory-confirmed cases against hospitalized cases. Differences in the pathogenicity and epidemiology of each strain in various environments further complicate the evaluation of the populations’ exposure to the virus in a specific area. To have a solid estimation of the degree of exposure of the population during each outbreak of the disease, a seroepidemiological investigation is very important. A relevant study was performed in the year 2010 in the epicenter of the outbreak in northern Greece. A weighted seroprevalence of 5.8% was estimated, and 18% of the people with WNV infection manifested symptoms attributable to this infection [6]. The results appeared similar to previous studies that had to do with WNV Lineage 1 (L1) outbreaks [14–16].

The aim of this study is to estimate the seroprevalence for WNV IgG antibodies in a representative sample from all over Greece after three consecutive years of WNV cases occurring in various geographical areas of Greece. By using seroprevalence data, the exposure of the population could be assessed together with a gross estimation of the pathogenicity of the virus. Moreover, the geographical distribution of positive sera may indicate circulation of the virus and the need for enhancing the surveillance system to capture clinical cases.

## Materials and Methods

### Study population—Sample collection

The population of the whole country was considered as the study population. The leftover sampling methodology was followed [17]. A geographically stratified sampling plan was applied to produce a representative sample using the strata as prefectures of Greece (NUTS-3). The required sample size was calculated to be about 4,000 blood samples ensuring the minimum sample for each spatial unit of NUTS-1 level (North Greece, Central Greece, Attica, Aegean islands-Crete) in order to make a comparison between NUTS-1 feasible and reliable. Taking into consideration an expected seroprevalence of 4.5%, a precision of 2% and a 95% confidence interval (CI) the minimum sample size for each area of NUTS-1 was calculated at 413 blood samples. As Aegean islands-Crete (the smallest NUTS-1) cover 10.5% of the total Greek population (Census 2001), the total sample was calculated at about 4,000 (413/0.105). Blood samples were collected from each stratum (prefectures) proportionally to its population size and age distribution, according to five age groups (0–24, 25–54, 55–64, 65–79, 80+).

During the 6-month period from the month of November 2012 to April 2013, a total of 3,962 leftover serum samples were collected from public and private laboratories (50 in total) from all prefectures of Greece according to the sampling schedule. From each participating laboratory we asked for a specific number of left over samples fulfilling the inclusion criteria (age, residence) according to the population of each region/prefecture. The samples belonged to people who used the laboratory services for various purposes: check-ups, other chronic diseases’ follow-ups *et cetera*. Both public and private laboratories provided information on age, sex, area of residence and occupation for each sample collected. The occupations were further grouped in five main categories: Farmer/herdsman/worker, housewife/unemployed, freelancer, child/student, retired.

### Diagnostic tools

A commercially available ELISA kit (WNV IgG DxSelect ELISA kit, Focus Diagnostics Inc, Cypress, CA) was used to detect IgG antibodies against WNV as previously described. The analysis was carried out in the Laboratory of Microbiology, Medical School, National Kapodistrian University of Athens, Greece and especially on the island of Crete at the Laboratory of Bacteriology, Parasitology, Zoonoses and Geographical Medicine of the University of Crete. The assay was performed according to the manufacturer’s instructions. Specimens with an index value of ≥ 1.50 were considered positive for WNV IgG antibodies. An index value of ≥ 1.30 and < 1.50 was considered as an equivocal result, while an index value of < 1.30 indicated that IgG antibodies were not detected. Similarly, a commercially available ELISA kit (WNV IgM DxSelect ELISA kit, Focus Diagnostics Inc, Cypress, CA) was used to detect anti-WNV IgM antibodies in IgG positive samples. Specimens with an index value of ≥ 1.10 were considered positive for WNV IgM antibodies. An index value of ≥ 0.90 and < 1.10 was considered an equivocal result, while an index value of < 0.90 indicated that anti-WNV IgM antibodies were not detected.

All the ELISA IgG positive samples were further tested by PRNT. PRNT was performed in a Biosafety Level (BL) 3 laboratory. Serum samples were inactivated by heat at 56°C for 30 minutes. A volume of 250 μL serum at 10% dilution were incubated with an equal volume of 200 plaque-forming units (PFU) per mL of WNV strain Egypt 101 for 75 minutes at 37°C in 5% CO_2_; 500 μL/well of the mixture was transferred into cells grown in 6-well plates that had been seeded 48 hours earlier, with Vero p cells (the medium was removed just before the transfer and washed 3 times). The plates were incubated for 1 hour at 37°C in 5% CO_2_. An overlay of 4 mL/well containing 2% Noble agar in culture medium with 2% fetal bovine serum and antibiotics (adjusted at 47°C) was added to the plates, which were incubated for 72 hours at 37°C in 5% CO_2_. A second 4 mL/well overlay was added with the difference that it contained 0.005% neutral red and the plates were incubated overnight at 37°C in 5% CO_2_. The number of plaques per well was assessed by virus back-titration. Serum was considered positive for anti-WNV antibodies when 90% reduction is seen compared with the virus control well, which should have about 100 plaques.

### Ethics Statement

Approval of the study protocol was received by the Ethics Committee of the University of Thessaly which waived the need for written consent. After verbal consent, anonymous left over serum samples were collected and sent to the Department of Hygiene and Epidemiology, School of Health Sciences, University of Thessaly, Greece.

### Statistical analysis

Statistical analysis was conducted taking the sampling plan into consideration through the procedure complex samples of SPSS. Pearson chi-squared test was used to evaluate the independence between categorical variables. The chi-square test was used as a measure of the association between WNV seropositivity and categorical variables. General Linear model was used to explore the linear association between ordinal variables and WNV seropositivity. The logistic regression model was used in the assessment of the impact of gender, age and area of residence on WNV seropositivity. P values <0.05 were considered statistically significant. Statistical analysis was performed using SPSS v21.0 (IBM Corp, Armonk, NY) and Stata v11 (Stata Corporation, Texas, USA). The Geographical Information Systems ARCGIS 10.1 (ESRI, Redlands, CA, USA) was used to map the WNV cases together with an area map of the seroprevalence on the prefecture level.

## Results

A total of 3,962 blood samples was collected through left over sampling from all over Greece according to the sampling protocol. Information about gender, age, area of residence (NUTS-1 and prefecture) and result of IgG seropositivity was provided for all participants. However, information about the professional status was available for 2,897 samples. Table 1 shows the sociodemographic characteristics of the sample.

**Table 1:** Study population characteristics.

Factors	N (%)
**Gender**	
Females	2543 (64.2)
**Age (years)**	
80+	119 (3.0)
65–79	543 (13.7)
55–64	439 (11.1)
25–54	1713 (43.2)
0–24	1148 (29.0)
**NUTS I (area)**	
North Greece	1136 (28.7)
Central Greece	1071 (27.0)
Attica	1354 (34.2)
Aegean Islands-Crete	101 (10.1)
**Profession**	
Employer in public/private sector	857 (33.9)
Farmer/Grazier/Worker	147 (5.8)
Freelancer	272 (10.8)
Housewife/unemployed	455 (18.0)
Child/Student	439 (17.4)
Retired	355 (14.1)

WNV IgG antibodies were detected in 82 samples while 61 were also positive in PRNT representing a weighted seroprevalence of: 2.1% (95% C.I.: 1.7–2.6) and 1.5% (95% C.I.: 1.2–2.0), respectively. Moreover, 5 out of the 82 IgG positive samples were also found positive for IgM antibodies. All the IgM positive samples were also PRNT positive. It is of interest that 9 positive PRNT samples came from seven prefectures (South Ionian, Thesprotia, Ioannina, Lakonia, Messenia, Fthiotida and Chania) where human clinical WNV cases were not recorded in the previous years.

As shown in Table 2, higher IgG seropositivity was found in people of older age (6.7%). Univariate analysis showed that age, area of residence and professional status were associated with WNV seropositivity. Similar results were found by using only the PRNT positive results with the exception of the area of residence, where the difference was not statistically significant. In multivariable analysis (Table 3), it was revealed that there was a significant association between IgG seropositivity, age (80+ vs. 0–24; OR = 3.72, 95% C.I.: 1.61–8.62) and area of residence (Attica vs. Aegean Islands-Crete; OR = 6.01, 95% C.I.: 1.46–24.79). When age was included as a dichotomous variable (65+ vs. <65 years), it was identified that people aged 65+ were more likely to be detected positive for IgG (OR = 3.47, 95% C.I.: 2.20–5.48). In sensitivity analysis, the professional status was added to the logistic regression model but it was not associated with IgG seropositivity. Gender, agricultural labor and retired status were not associated with WNV seropositivity. Similar results were discovered by using the PRNT positive results as the outcome variable (Table 3).

**Table 2:** West Nile Virus seropositivity in comparison to gender, age, area of residence, occupation and retired status (Univariate analysis).

Factors	Positive for WNV					
	n/N	%	95% C.I.	RR	95% C.I.	p-value
**Gender**						
Male	35/1419	2.5	1.8–3.4	1.34	0.87–2.06	0.189
Female	47/2543	1.8	1.4–2.5			
**Age (years)**						
80+	8/119	6.7	3.4–12.9			0.001
65–79	25/543	4.6	3.1–6.7			
55–64	5/439	1.1	0.5–2.7			
25–54	22/1713	1.3	0.8–1.9			
0–24	22/1148	1.9	1.3–2.9			
**Age (years)**						
65+	33/662	5	3.6–6.9	3.36	2.18–5.18	<0.001
<65	49/3300	1.5	1.1–2.0			
**NUTS I (area)**						
North Greece	26/1136	2.3	1.6–3.3			0.040
Central Greece	18/1071	1.7	1.1–2.6			
Attica	36/1354	2.7	1.9–3.7			
Aegean Islands-Crete	2/401	0.5	0.1–2.0			
**Occupation**						
Employer in public/private group	14/857	1.6	1.0–2.7			0.002
Farmer/ grazier/ worker	3/147	2.0	0.7–6.1			
Freelancer	1/272	0.4	0.1–2.6			
Housewife/ unemployed	6/455	1.3	0.6–2.9			
Child/student	17/811	2.1	1.3–3.3			
Retired	17/355	4.8	3.0–7.6			
**Retired**						
Yes	17/355	4.8	3.0–7.6	2.97	1.70–5.17	<0.001
No	41/2542	1.6	1.2–2.2			

**Table 3:** Results of the logistic regression model used for the assessment of gender, age and NUTS I-level area of residence on WNV seropositivity.

Factors	ELISA			PRNT		
	OR	95% C.I.	p-value	OR	95% C.I.	p-value
**Gender**						
Male	1.35	0.85–2.12	0.201	1.05	0.62–1.79	0.858
Female	1.00			1.00		
**Age**						
80+	3.72	1.61–8.62	0.002	4.69	1.74–12.64	0.002
65–79	2.48	1.39–4.43	0.002	3.85	1.93–7.67	<0.001
55–64	0.59	0.22–1.57	0.292	0.80	0.26–2.46	0.691
25–54	0.67	0.37–1.22	0.193	0.76	0.36–1.59	0.466
0–24	1.00			1.00		
**NUTS I (area)**						
North Greece	4.91	1.17–20.57	0.029	3.18	0.74–13.58	0.118
Central Greece	3.35	0.78–14.39	0.104	2.67	0.61–11.67	0.191
Attica	6.01	1.46–24.79	0.013	4.05	0.97–16.91	0,055
Aegean Islands-Crete	1.00		.	1.00		.

The ratio of WNND patients to infected persons, considering only the population in affected areas, was found to be 1:376 (95% C.I.: 1:421–1:338), (Table 4). This ratio increased with age, and almost doubled in persons aged 65 years or older. More specifically, it was 1:501 (95% C.I.: 1:397–1:584) in those aged 25 up to 64 years old, and 1:266 (95% C.I.: 1:217–1:326) in those aged 65 years or older. A similar increase with age was found when the ratio of WNND patients to infected persons using PRNT results (Table 4). Moreover, as presented in Table 5, the highest ratio was found in Northern Greece while the lowest was found in Aegean Islands-Crete.

**Table 4:** Ratio of West Nile neuroinvasive disease (WNND) to infection by age-group.

Age (years)	WNND cases	Population in study area	Ratio of WNND to infection (95% CI)	Population in affected areas	Ratio of WNND to infection (95% C.I.) (of affected areas)—ELISA	Ratio of WNND to infection (95% C.I.) (of affected areas)—PRNT
**80+**	84	583,334	1:465 (1:724, 1:296)	355,156	1:283 (1:440, 1:180)	1:213 (1:379, 1:109)
**65–79**	182	1,525,518	1:385 (1:653, 1:302)	1,061,744	1:268 (1:338, 1:210)	1:247 (1:322, 1:187)
**55–64**	47	1,286,137	1:301 (1:556, 1:186)	764,510	1:179 (1:330, 1:111)	1:148 (1:303, 1:64)
**25–54**	54	4,671,656	1:1125 (1:1260, 1:921)	3,124,653	1:752 (1:843, 1:616)	1:506 (1:654, 1:392)
**0–24**	11	2,749,641	1:4749 (1:6509, 1:4051)	1,470,553	1:2540 (1:3485, 1:2167)	1:1513 (1:1691, 1:1481)
**All ages**	378	10,816,286	1:601 (1:673, 1:539)	6,776,616	1:376 (1:421, 1:338)	1:276 (1:321, 1:236)

**Table 5:** Ratio of West Nile neuroinvasive disease (WNND) to infection by NUTS I area.

NUTS I (area)	WNND cases	Population in study area	Ratio of WNND to infection (95% C.I.)
**North Greece**	272	3,110,835	1:263 (1:335, 1:207)
**Central Greece**	53	2,745,706	1:881 (1:1032, 1:759)
**Attica**	51	3,828,434	1:2027 (1:2114, 1:1915)
**Aegean Islands-Crete**	2	1,131,311	1:2828 (1:4673, 1:3130)

**Fig 1:**
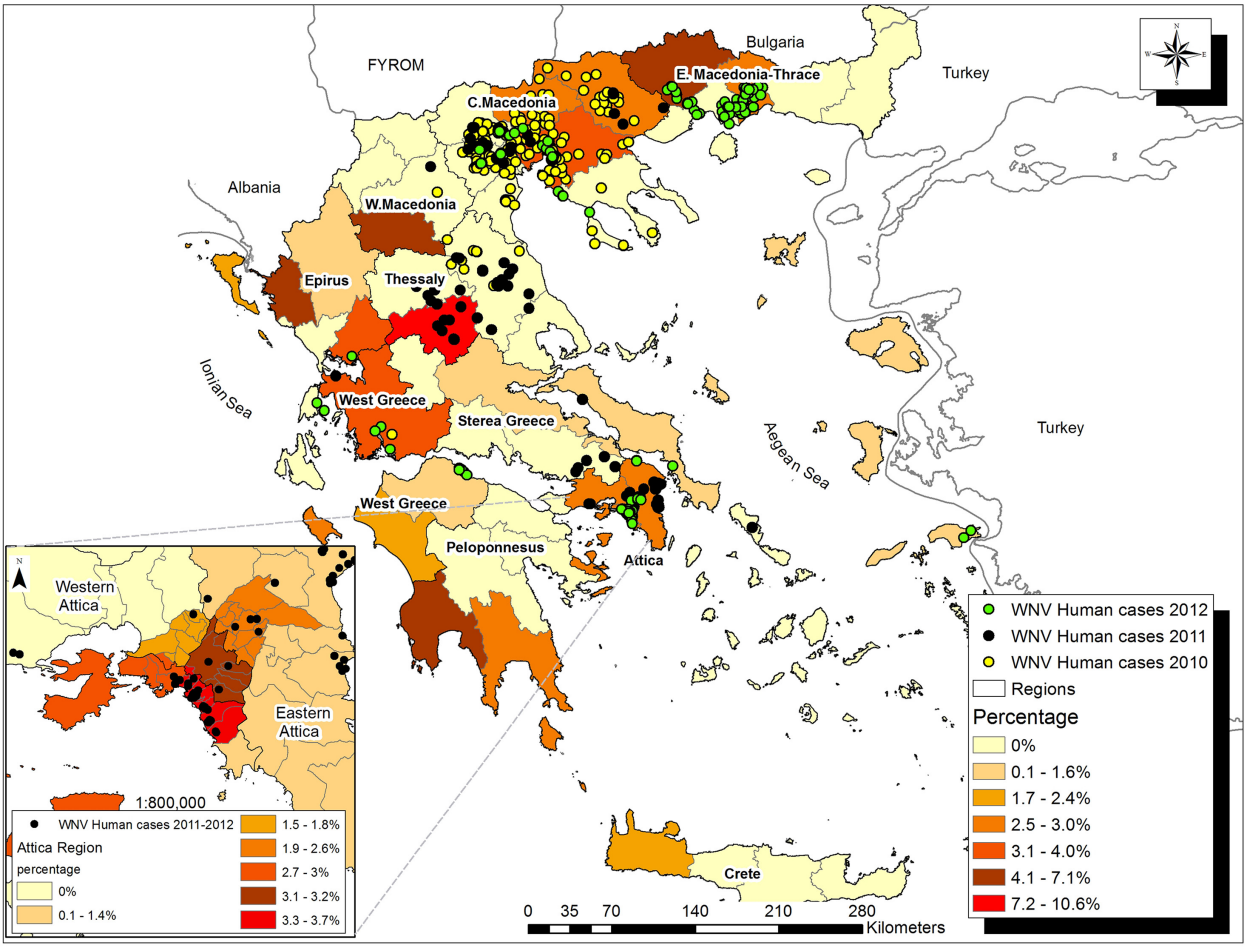
Area map of Greece according to WNV IgG seroprevalence levels (level NUTS 3) together with the geographical distribution of recorded human cases recorded during 2010–2012.

The seroprevalence differed among the geographical regions of Greece, with 25 out of 52 (48.1%) prefectures exhibiting seroprevalence of 0%. Significantly higher seroprevalence were detected in the prefecture of Karditsa (10.6%, 95% C.I.: 1.82–19.45), Thessaloniki (4.0%, 95% C.I.: 2.00–5.93), Central Attica (3.2%, 95% C.I.: 1.49–4.91) and South Attica (3.7%, 95% C.I.: 1.00–6.36), (Fig 1). Seven prefectures had seropositive samples (both ELISA and PRNT) without any recorded human case (Table 6).

**Table 6:** Geographic areas with positive samples by both ELISA IgG and PRNT and no recorded human cases.

Prefecture	Total number of samples	Positive samples	Percentage of positive samples	95% C.I.
**South Ionian**	36	1	2.8%	0.0–8.14
**Thesprotia**	17	1	5.9%	0.0–17.00
**Ioannina**	62	1	1.6%	0.0–4.75
**Lakonia**	36	1	2.8%	0.0–8.14
**Messenia**	65	4	6.2%	0.31–11.99
**Fthiotida**	65	1	1.5%	0.0–6.53
**Chania**	55	1	1.8%	0.0–5.35

## Discussion

In our study we estimated a seroprevalence of 2.1% in ELISA IgG and 1.5% in PRNT in a nationwide stratified sample according to their geographic regions and age groups. This percentage is similar to that identified in other endemic areas (New York (2.6%, 95% C.I.: 1.2–4.1) [13]), or countries: northern Italy (2.08%) [18], southeastern France (1.42%) [19] and in recent studies in Turkey [20,21]. A lower percentage of samples (0.13%) was found with IgM antibodies representing recent infection. This is in accordance with the recorded number of WNV cases which was higher during the year 2010 (262 cases) in comparison with 2011 (100 cases) and 2012 (161 cases).

In Greece, a survey using Immunofluorescence Assay (IFA) that was conducted in the year 1990, showed that WNV or an antigenically related flavivirus was active in Greece, since ~1% of the population in specific areas of Greece (Northern and Southern Greece) was positive for WNV IgG antibodies [22]. Higher seroprevalence of antibodies was estimated in the year 2010, within the epicenter of the outbreak in the region of central Macedonia (5.8%, 95% C.I.: 3.8–8.6) [6], while the seroprevalence estimated in our study, present in the same region, but not within the epicenter was lower (2.8%, 95% C.I.: 1.6–4.1).

The regions with high number of human cases presented high seropositivity, but statistical analysis failed to show any significant association between them. One important finding of the study is the identification of seven prefectures (South Ionian, Thesprotia, Ioannina, Lakonia, Messenia, Fthiotida and Chania) with confirmed seropositive samples in which no human cases were recorded in the past years. In the retrospective review of all the hospitals serving one of these regions (Messinia), we identified a number of cases fulfilling the clinical criteria of WNV: fever and encephalitis or meningoencephalitis. These identified cases were hospitalized, but one died,aand they were never tested for WNV infection. Thus, it is quite possible that human cases of WNV occurred within these regions but they were never diagnosed or reported. For this reason, we calculated the ratio of seropositivity to human WNND cases in the whole country, excluding the above regions with seropositive results that the void of human cases. We identified a ratio of 1 neuroinvasive case per 376 ELISA IgG seropositive persons at all ages. This ratio is considered lower than that estimated in two previous studies (1:141 [6], 1:140 [13]) and the main reason for this, is the difference in the methodology adopted in these studies. The previous studies were designed and conducted at the epicenter using questionnaires and interviews, while in our study we used national data from mandatory notification. Our results may have been affected by underdiagnoses and underreporting of WNV cases. It has to be noted that in other studies carried out in Israel, Italy and the USA, which applied a methodology that is more similar to that of our study, the ratio of neuroinvasive cases ranged between 1:200 and 1:400 [15,18,23] which is closer to our estimation. However, considering the 1:376 ratio in the seven prefectures with no human cases, we estimated that a total of 68 (range 61–76) WNV infection cases may have been missed. If we take into consideration the total number of WNV diagnosed and reported cases (381) within the period of the year 2010–2012, then, in these seven prefectures the underdiagnosis rate rises up to 17.8%. We have to acknowledge that this estimation is not accurate taking into consideration, among other factors, the possibility of someone being exposed to mosquitoes in a different region of the area of residence. It is also of interest to point out that in 15 out of 52 prefectures we calculated zero seropositivity with no recorded human WNND cases. In the next two transmission periods (2013 to 2014), new cases of WNND occurred in two out of the 15 prefectures with previous zero cases and seropositivitty. After this geographic expansion of WNV, we can assume that virus will continue to expand and affect new geographic areas as it happened in the USA [24,25]. If in the future we continue to have non endemic areas for WNV, a detailed ecological and environmental study among affected and non-affected areas would give more information about the epidemiology of the disease in Greece and may help in the control activities. Moreover, we can enhance the surveillance of human cases in order to verify the non-existence of cases in these areas and a second serosurvey in due time could capture any changes in the epidemiology of WNV in Greece.

In our study a significant association between age and WNV seropositivity was identified. Evidence of higher probability of exposure among older people was found in previous seroprevalence studies either in Greece [6] or in other countries such as Israel, Spain, and Egypt [15,26,27]. The past circulation and exposure of the human population to WNV [6], the time spent outdoors (for example, agricultural activities) [16], the less frequent use of insect repellents, the measures to adopt to protect people against mosquito bites/exposure, and the inadequate knowledge and education level were suggested as possible explanations [13]. Old people (>80) seem to have a lower ratio (1:283) of infection suggesting a higher risk of developing WNND, which is a common finding in previous studies from both Greece and the USA [6,13,21]. The overall data of both the present study along with those of previous studies indicated that the elderly people are more prone to be infected and to develop WNND, suggesting that the age above 65 should be a target group for prevention campaigns among the general population and the health professionals.

Despite findings from previous studies in our statistical analysis, the professional status (agricultural labor), gender, and retired status was not associated with seropositivity [16,28].

The left over methodology of sampling has the advantage of being of low cost and of sufficient representativeness but does not include randomization. Thus, the representativeness of our sample could be diminished despite the fact that we stratified the sample according to the geographic area of residence, sex and age group. Moreover, we do not have information about the non-responders and non-participants to enable us assess for selection bias. However, this methodology has been used successfully in seroepidemiological studies of vaccine-preventable diseases and it is a prominent tool used to conduct serosurveillance [17]. Another limitation of our study is the lack of information related with the exposure to mosquitos or other preventive measures. It should be noted that the samples were collected from persons who do not use the laboratory for the diagnosis of WNV, since the diagnosis of WNV in Greece is conducted only in two reference laboratories. Thus, possible overestimation of the seroprevalence is not expected. In this study, we did not test the samples for Usutu virus to eliminate the possibility of cross reactivity with WNV antibodies. However, till date, no data has been available to prove that the Usutu virus is present in wild birds of Greece, despite the active search by the Department of Microbiology and Parasitology of the Faculty of Veterinary Medicine, University of Thessaly. Between years 2009–2014, a total of 800 avian serum and tissue samples from wild birds were examined serologically by polymerase chain reaction (PCR) and the presence of Usutu virus was not documented [29]. The Usutu virus was documented serologically in one pigeon among 247 pigeons that were found to be positive for WNV in 2011 [30]. Thus, even if Usutu virus is present in Greece, the circulation of the virus could be considered very limited and cannot affect our results substantially.

In conclusion, our results support the establishment of WNV in Greece with a seroprevalence of 2.1% (1.5% with PRNT) in a nationwide sample, after three consecutive years with WNV cases. Moreover, elderly people are at higher risk both to get infected and to develop WNND. In some regions positive serum resulting in the absence of recorded human WNV cases, indicate possible underdiagnosis and underreporting of the disease. Preventive measures should focus more on elderly people especially those in endemic regions.
